# Family planning service availability and readiness assessment of primary health care facilities in Delta State, Nigeria: a mixed methods survey

**DOI:** 10.1186/s12978-023-01693-x

**Published:** 2023-10-24

**Authors:** Dorcas Tom Obong, Patrick Oyibo

**Affiliations:** 1 Department of Community Medicine, Delta State University Teaching Hospital, Oghara, Nigeria; 2 Health Services Research and Management Division, School of Health and Psychological Sciences City, University of London, London, UK; 3 Department of Community Medicine, Faculty of Clinical Medicine, College of Health Sciences, Delta State University, Abraka, Nigeria

**Keywords:** Contraceptives, IEC materials, Primary health facilities, Family planning readiness, Service availability

## Abstract

**Background:**

The availability of contraceptives, family planning guidelines, and Information, Education, and Communication (IEC) materials can increase access to family planning services. This study assessed the availability of commodities and readiness of primary health care (PHC) facilities in Delta State to offer family planning services.

**Methods:**

A cross-sectional design with an explanatory mixed-method approach was used i.e., the authors first collected the quantitative data, and after preliminary analysis of quantitative information, the qualitative approach was utilised to gather data on the perspectives of 32 PHC facility managers and 6 reproductive health supervisors on factors affecting family planning service availability and readiness.

**Results:**

Twenty-one (65.6%) of the PHC facilities surveyed offered at least five modern methods of family planning. Stock-outs of emergency contraceptives, implants, intra-uterine contraceptive device (IUCD), oral contraceptive pills (OCP), condoms, and injectables were observed in 31 (96.9%), 17 (53.1%), 13 (40.6%), 4 (12.5%), 2 (6.3%), and 1 (3.1%) of the facilities respectively. Eleven (34.4%) and 8 (25.0%) of the facilities had IEC materials and family planning guidelines, and contraceptive commodity checklists respectively. Seventeen (53.1%) of the facilities did not have complete records of family planning activities.

**Conclusion:**

This study shows that a significant proportion of PHC facilities had stock-outs of contraceptive commodities, no complete records of contraceptive activities, no IEC materials and no family planning checklists. Continuous training of health providers and increased government commitment can help to improve contraceptive services.

## Introduction

Family planning refers to the use of modern contraception and other methods of birth control to regulate the number, timing and spacing of human births [1]. The implication of this is that parents, particularly mothers, can plan their lives without being overly subject to sexual and social imperatives [1, 2]. It reduces maternal and child mortalities, reduces the number of unsafe abortions, prevents sexually transmitted infections, enhances women empowerment, and helps in population control [3–5]. However, despite these impressive gains from family planning, most low- and middle-income countries have consistently recorded high fertility rates and low contraceptive prevalence rates (CPR) [6, 7]. Amongst the numerous reasons for these poor indices, is the non-availability of contraceptives in health facilities which limits women’s access to family planning services [8].

Globally, the number of women desiring to use family planning has increased markedly over the past two decades, from 900 million in the year 2000 to nearly 1.1 billion in 2020 [9]. These women would need to have access to contraceptives. Similarly, in developing regions, an estimated 218 million women who want to avoid pregnancy are not using contraceptives for many reasons including contraceptive unavailability [10]. Family planning service availability and readiness is essential to achieving the sustainable development goal (SDG) of universal access to sexual and reproductive health including family planning by 2030 [11]. Therefore, it is important that health facilities have a reliable supply of a broad mix of family planning commodities made accessible to consumers, thereby making it easier for women of reproductive age to choose a method they like and switch methods when they want [12–15]. Thus, when there are problems with the supply chain, resulting in stockouts or temporary unavailability of contraceptives at a health facility, this may result in women losing faith in the health service and discontinuing use of contraception [12]. Other factors affecting contraceptive availability include poor knowledge of contraceptives among providers, lack of trained staff, lack of equipment and low demand from clients [16, 17]. To ensure universal coverage of family planning services, health facilities should be ready to always provide these services. The current study was conducted with the aim of assessing the family planning service availability and readiness in primary health care facilities in Delta State, Nigeria. Findings from the study will provide evidence to inform policy decision making by the relevant authorities concerned with the provision of family planning services in the study setting and other similar settings in Nigeria.

## Methods

### Study setting and design

The study was carried out from November 2017 to January 2018 in thirty-two randomly selected primary health care facilities in Delta State, Nigeria. The State is one of the 36 States in Nigeria, and it is situated in the oil-rich Niger Delta region. The geographical area of the State is divided into upland and riverine with twenty-five Local Government Areas (LGA) categorized into three senatorial districts namely Delta North, Delta Central and Delta South. The population of women of reproductive age (15–49 years) in Delta State was 901,646 as at the year 2011 [18]. The literacy level in the State is 65.7% (males—34.5% and females—31.2%) [19]. Most of the PHCs offer family planning services. A cross-sectional design with mixed (quantitative and qualitative) methods approach was employed to assess the family planning services availability and readiness in primary health care facilities. The authors first collected the quantitative data, and after preliminary analysis of quantitative information, the qualitative approach was utilized to gather data on the perspectives of PHC facility managers and reproductive health supervisors on factors affecting family planning service availability and readiness.

### Study population

The study population comprised of representative sample of thirty-two primary health care facilities selected from six LGA (two per senatorial districts), thirty-two primary health care facility managers in the selected PHC facilities and six reproductive health supervisors in the six selected LGAs.

### Sampling technique

A multi-stage sampling technique (two stages) was employed in this study. In the first stage, a simple random sampling technique (balloting) was used to randomly select two LGAs from each of the three senatorial districts, giving a total of six LGAs. In the second stage, a sampling frame consisting of all PHC facilities in the six selected LGAs was obtained. Facilities offering family planning services were identified and 50% of those facilities were selected by simple random sampling (table of random numbers) giving a total of thirty-two PHC facilities. For the qualitative study, thirty-two PHC facility managers and six reproductive health supervisors (RHS) were purposively selected.

### Study instruments

A pretested standard observational checklist, key informant interview guide, and an in-depth interview guide were the study instruments utilized. The observational checklist was adapted from WHO and USAID service availability and readiness assessment (SARA) reference manual (version 2.2 July 2015) [20]. The checklist consisted of five sections used to obtain quantitative data on the availability of family planning guidelines, Information, Education and Communication (IEC) materials, and the validity and stock-outs of contraceptives.

The in-depth interview guide was adapted from operation research on task shifting in the provision of contraceptive implants conducted in Northern Nigeria [21]. It consisted of two sections used to obtain qualitative data on factors affecting contraceptive availability at the PHC facility level. The key informant interview guide for the reproductive health supervisors was developed by the principal researcher to elicit more qualitative data on factors affecting commodity availability at the local government level.

### Data collection

On the scheduled days of visit to the different health facilities, trained data collectors inquired from the PHC facility managers about the availability of family planning commodities; family planning guidelines; family planning checklists; family planning records; and IEC materials. Each PHC facility manager had to provide evidence of availability of all the listed items. The trained data collectors also checked the validity of available contraceptives through the expiry dates. The daily consumption register was also examined for stock-outs and evidence of proper and complete documentation.

### Qualitative data collection

In-depth and key informant interviews were conducted with the PHC facility managers and the RHS respectively. Both in-depth and key informant interviews lasted for about 30 min. Qualitative data obtained were audio taped with the consent of the participants.

### Outcome variables

The outcome variables were the proportion of facilities offering at least five modern methods of contraceptives; proportion of facilities with family planning guidelines, checklists, IEC materials, and complete records; and proportion of facilities with stock-outs of contraceptives. The proportion of facilities offering at least five methods was determined using the criteria adapted from the Global programme on reproductive health commodity security which set the cut-off for available commodities at five. [22].

### Statistical analyses

The statistical package for social sciences software (SPSS) version 21 (IBM SPSS Inc. Chicago. IL, USA) was used for statistical analysis. Each questionnaire was sorted, coded, and validated before entering into the IBM SPSS version 21 spreadsheet. The proportion of health facilities with available valid contraceptive commodities was presented using a table. The proportion of facilities having IEC materials, guidelines, stockouts and complete records of family planning commodities were presented using charts. Qualitative data recordings were transcribed, coded, and analyzed using content thematic analysis. In some cases, some of the reported statements by the interviewees were quoted verbatim.

### Ethical considerations

The Health Research Ethics Committee (HREC), Delta State University Teaching Hospital provided ethical clearance for the study (HREC/PAN/2017/008/0234). The study conducted followed relevant guidelines and regulations. Approval for the study to be conducted was obtained from the Delta State Local Government Service Commission, and the head of department for health in each selected LGA. Informed consent was obtained from the PHC facility managers and the reproductive health supervisors in each selected LGA.

## Results

### Quantitative findings

Injectable contraceptives were available and valid in 31 (96.9%) health facilities, while 28 (87.4%), 27 (84.4%), 18 (56.3%), and 13 (40.6%) of the surveyed health facilities had valid condoms, oral contraceptive pills (OCP), IUCD, and implants respectively. Emergency contraceptives were available and valid in only one (3.1%) health facility (Table 1).

**Table 1:** Availability and Validity of contraceptives in the surveyed health facilities

	Contraceptive methods (N = 32)					
	OCP	Injectables	Condoms	IUCD	Implants	Emergency contraceptives
Contraceptives availability and validity						
Available and valid (not expired)	27 (84.4)	31 (96.9)	28 (87.4)	18 (56.3)	13 (40.6)	1 (3.1)
Available, not valid (expired)	1 (3.1)	0 (0.0)	2 (6.3)	1 (3.1)	2 (6.3)	0 (0.0)
Not available	4 (12.5)	1 (3.1)	2 (6.3)	13 (40.6)	17 (53.1)	31 (96.9)

**Fig. 1:**
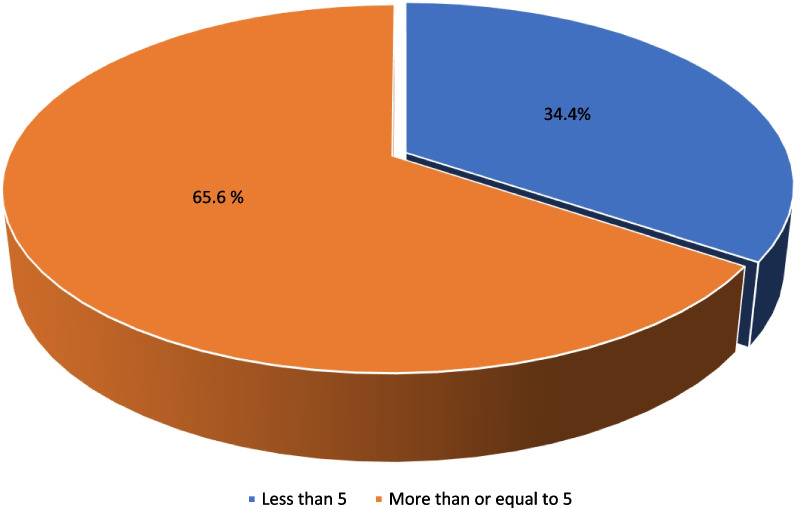
Proportion of health facilities offering at least five modern methods of family planning on day of assessment

Twenty-one (65.6%) of the health facilities surveyed offered at least five modern methods of family planning (Fig. 1). Stock-outs of emergency contraceptives, implants, IUCD, OCP, condoms, and injectables were observed in 31 (96.9%), 17 (53.1%), 13 (40.6%), 4 (12.5%), 2 (6.3%), and 1 (3.1%) of the surveyed health facilities respectively (Fig. 1).

**Fig. 2:**
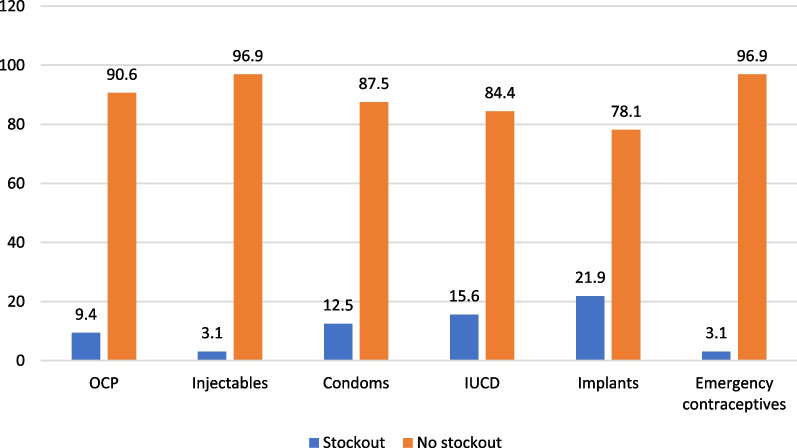
Bar chart showing the proportion of health facilities with stock outs of the various contraceptive methods

On the day of survey, 8 (25.0%) and 11 (34.4%) of the health facilities had contraceptive commodity checklists and IEC materials, and family planning guidelines respectively. Seventeen (53.1%) of the health facilities did not have complete records of family planning activities (Fig. 2).

### Qualitative findings

**Fig. 3:**
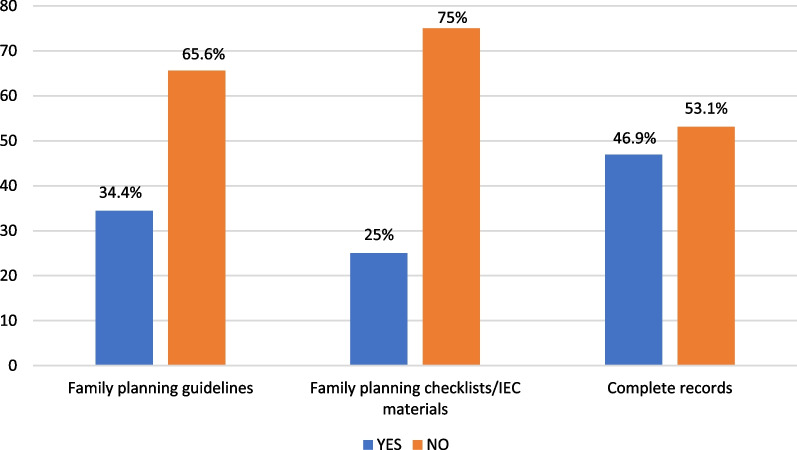
Proportion of health facilities with available family planning materials and complete records on day of assessment

To triangulate quantitative findings, key qualitative results from both IDIs and KII were summarised to themes that emerged during discussion. The major factors or themes that emerged were the low demand by clients for contraceptives, inadequate training of staff, stock-outs of contraceptives, and unavailability of services on weekends. Some of the facility managers spoke extensively about these themes (Fig. 3).

### Low demand by clients

Most of the respondents rated the demand for family planning among women as being poor. However, a few said the demand was for specific commodities as one respondent stated:

*“The women are not coming. They have so many false beliefs and misconceptions*. *The few that come around requests for only injectables and condoms.”* (51-year-old nurse).

### Inadequate training of health providers

 Most of the respondents stated that training of staff to deliver family planning methods has not been a priority for government. Staff do not undergo regular training on contraceptives, and this has resulted in low staff confidence in counseling and administering certain contraceptives. A respondent stated:

“*As far as counseling is concerned some nurses are more confident than the CHEWS, however, it depends on their level of training or exposure.”* (56-year-old Nurse).

A few respondents acknowledged the significant role of non-governmental organizations like Marie Stopes in staff training. A respondent stated:

*“Before now, the government trained all staff, but in recent times only the focal person for family planning has been trained, who will then step down when she comes back from such training. We were thinking they would come back and pick others to train but we are yet to see them”*. (48-year-old* nurse).*

Another respondent said, *“I have been working for a long time, only Marie Stopes has trained me. I have not gone for any training organized by the State government.”* (48-year-old RHS).

### Stock outs of family planning commodities

Majority of the respondents said that they get their supply of family planning every 2–3 months. However, in recent times, the supply has been interrupted resulting in periods of stock-outs ranging from 6 to 18 months. One respondent said:

“*Initially we were getting supplies every 2 months that was like 4–5 years ago, but since 2015 supply has been irregular from the State. There was no supply from the central stores. Sometimes transporting them to the various LGA is a big problem. The last time we received commodities was 4 months ago and they brought more than enough for the LGA, we don’t know when they will come again” (*49-year-old RHS).

Another said: “*We had stock outs for about 19 months from March 2015 to September 2016. It was really a difficult time. The nurses made contributions among themselves to buy some commodities and we charged the clients for it”* (49-year-old nurse).

Another respondent said, *“I did not have any supply of family planning commodities for 18 months. I had to discuss with a nearby pharmacy shop to subsidise the commodities for us. We tell the clients that government has not supplied and that is why they are paying for it.”* (48-year-old RHS).

### Unavailability of services on weekends

Most of the respondents when asked on the availability of family planning services in their health facility stated that the service was available all days of the week including weekends despite having a fixed day for the clinic. However, for facilities that open, not all services are given as one respondent noted: “*We do not provide implants on weekends because the staff that was trained on this method does not work on weekends.”* (40-year-old nurse).

A respondent noted that security concerns were a huge setback, she said: *“We do not open on weekends, because of security reasons. This place is usually very quiet during weekends compared to weekdays and the local government has not given us any form of security.”* (57-year-old Nurse).

## Discussion

The analysis of this study revealed that despite renewed efforts to scale up family planning services in Nigeria, the availability of some contraceptives especially the Long-Acting Reversible Contraceptives (IUCD and Implants) was still low in most health facilities. These contraceptives require higher levels of skill and training to be able to administer them. The implication of this is that lower cadre health providers, or those who lack sufficient training and skills needed to administer these commodities, would make no effort to have them in stock. In this study, reproductive health supervisors attributed the low availability of contraceptives to lack of supply from the central stores in the State leading to stock-outs of commodities and thus affecting the contraceptives methods mix in the health facilities. This observation corroborates with findings from a previous study conducted in Nepal [22].

The three most common contraceptives observed in this study were the injectables, condoms and OCP in decreasing order and has been reported by previous similar studies conducted in Ghana and Nigeria [23, 24]. Discussions with the facility managers reveal that contraceptive availability depends on demand by clients hence, contraceptives with a higher demand would be the most available. Thus, from this study, injectable contraceptives had a higher demand than other contraceptives. This is mostly because of its ease of administration.

This study also observed that only one health facility had emergency contraceptives. This is a bit worrisome considering the fact that the need for emergency contraception exists throughout societies where prevalence of unintended pregnancies remains high, especially with widespread incidents of gender-based violence, rape, conflict, humanitarian disasters, refugee and displacement situations, as well as other emergencies as is the case in Nigeria today [21]. For women caught in these situations, emergency contraception represents perhaps the only chance to prevent a pregnancy that is not wanted [25]. Therefore, availability of emergency contraceptives is an indication of health facility readiness to offer family planning services.

The global programme on reproductive health commodity security under the UNFPA recommended availability of at least 5 modern contraceptives in a health facility to allow for a broad method mix and increase the chances of a woman getting her preferred method [22]. The modern methods under consideration were the condoms, OCP, injectables, IUCDs, implants and sterilization. In this study, it was found that a little above one-third of the surveyed health facilities did not offer up to five modern contraceptive methods. A similar study conducted in some PHCs in Nigeria revealed that a quarter of facilities did not have up to three contraceptives available [26]. The proportion of health facilities not offering up to five modern contraceptive methods observed in this study was lower compared to the report from a previous study conducted in Nepal where sixty-five percent of the PHCs did not offer at least five modern contraceptive methods [22].

None of the health facilities surveyed in this study offered sterilization as a contraceptive option. This is because sterilization requires a higher level of skill and a more developed infrastructure for its provision which is not available in primary health facilities. Apart from this, evidence has revealed that women hardly request for sterilization because they generally perceive sterilization as a method that one would select only out of necessity such as in situations where another pregnancy could threaten their lives or when other methods had failed. Many believe that if a couple has no medical issues, they should not consider sterilization until they have had many children, the woman is nearing menopause, or the man is very old [27]. To meet the varying needs and demands for contraception, sterilization for both males and females should be promoted for couples who have achieved their desired family size. Clearly, this will involve making these methods more accessible and ensuring that qualified personnel are available to provide them [28].

In this study, stock-outs of contraceptives varied by method type with Long-Acting Reversible Contraceptives (IUCD and Implants) being more stocked out. Similar findings from previous studies conducted in Nigeria [26] and Nepal [29] revealed that about half of PHC facilities recorded ‘stock-out’ of contraceptives. The observation from this study is most worrisome because many women now prefer to use these Long-Acting Reversible Contraceptives (IUCD and Implants) due to its effectiveness and longer protection. These could have implications for clients’ satisfaction and continuation of family planning use. Thus, methods offered at health facilities must be consistently available to ensure that there are no gaps in supply of desired contraceptives and to achieve a CPR of 68 percent by the year 2030 [30]. One factor identified in this study as causing stock-outs is poor documentation. Less than half of the surveyed health facilities had complete records of contraceptive supplies making it difficult to know when there are actual stock-outs of commodities. Accurate reporting helps family planning providers and logistic system managers to keep track of commodities used and helps to determine when to make requisitions [12]. It also serves as a monitoring and evaluation tool for the programme [31]. Forgetfulness on the part of health providers, lack of time, work overload and non-availability of documentation materials can result in poor documentation [32]. Discussions with the facility managers revealed that regular training for health providers has been deficient. This therefore calls for urgent steps by relevant stakeholders to train health providers regularly.

To ensure health facilities’ readiness for family planning services, it is important to consider contraceptive validity (expiration). Contraceptives should not only be available but should be valid such that a client can walk into a health facility requesting for her method of choice and get it delivered to her. This study showed that about a fifth of the surveyed health facilities had invalid (expired) contraceptives. This means that a substantial number of contraceptives would be unavailable for use by clients. This observation contrast with findings from a previous study conducted in Ghana and Armenia which reported that all the contraceptives studied were available and valid in all the public health facilities surveyed [33, 34]. This therefore calls for urgent action on the part of relevant stakeholders to ensure that contraceptives are not only available but valid.

Regarding the availability of family planning guidelines and other materials, a substantial number of the facilities recorded few or non-availability of these essential materials. This observation was similar to what was obtained in Edo State, Southern Nigeria, where contraceptive commodities and materials were available in three-quarters of the Primary health centres assessed [35] but differed from that of a study conducted in Lesotho where flip charts or posters were available in 84 percent of facilities, while brochures, pamphlets and other IEC materials were available in 65 percent of the facilities [36]. IEC materials are visual aids which if conspicuously displayed within the health facility contributes to the effectiveness of communicating family planning messages to clients. It also helps providers improve on their knowledge and quality of services rendered thus contributing to the overall uptake of family planning services [37].

A limitation of this study was the small sample size which may affect generalizability of findings from this study. Subsequent studies on this will endeavor to improve on this.

In conclusion, this study showed that the most available contraceptives were the injectables, condom and OCP in decreasing order with majority of the health facilities having up to 5 commodities. This study also revealed that stock-outs of IUCD and implants were observed in a quarter and less than a third of the facilities respectively. Over half of the facilities had no complete records of contraceptive activities while IEC materials and checklists were available in a quarter of the facilities. We recommend increased commitment by the relevant stake holders saddled with the responsibility of providing family planning commodities in all health care facilities. There should be training of family planning for doctors, nurses, community health extension workers. This will ensure that all health workers graduate with adequate skill in counselling and provision of common methods of contraception. In-service training for implants and IUCD insertion should be encouraged for every staff working in a PHC facility. There should be adequate budgetary allocation for family planning as well as timely release of funds to ensure procurement of commodities and improve logistics services.

## Data Availability

The datasets generated and/or analysed during the current study are not publicly available due to the reason that it was gathered for the postgraduate fellowship research of the first author by the first author but are available from the first author on reasonable request.
